# Influence of vaccination on critical COVID-19 patients with acute respiratory failure: a retrospective cohort study

**DOI:** 10.1186/s40001-024-01840-5

**Published:** 2024-04-20

**Authors:** Hsiao-Chin Shen, Jhong-Ru Huang, Chuan-Yen Sun, Ying-Ting Liao, Hung-Jui Ko, Chih-Jung Chang, Jia-Yih Feng, Yuh-Min Chen, Wei-Chih Chen, Kuang-Yao Yang

**Affiliations:** 1 Department of Chest Medicine, Taipei Veterans General Hospital, #201, Sec. Shih-Pai Road, Taipei, 11217 Taiwan; 2 School of Medicine, National Yang Ming Chiao Tung University, Taipei, Taiwan; 3 Institute of Emergency and Critical Care Medicine, National Yang Ming Chiao Tung University, Taipei, Taiwan; 4 Cancer and Immunology Research Center, National Yang Ming Chiao Tung University, Taipei, Taiwan; 5 Division of Evidence-based Medicine, Department of Medical Education, Taipei Veterans General Hospital, Taipei, Taiwan

**Keywords:** Acute Respiratory Failure, Acute Respiratory Distress Syndrome, Coronavirus Disease 2019 (COVID-19), COVID-19 Vaccines, Virus Shedding

## Abstract

**Background:**

Despite vaccines’ effectiveness in reducing COVID-19 infection rates and disease severity, their impact on critical patients presenting with acute respiratory failure is elusive. The aim of this study was to further investigate the influence of vaccination on mortality rates among severely ill COVID-19 patients experiencing acute respiratory failure.

**Methods:**

This retrospective cohort study was carried out at a tertiary medical center in Taiwan. From April to September 2022, patients who tested positive for the severe acute respiratory syndrome coronavirus 2 (SARS-CoV-2) through reverse transcription polymerase chain reaction (RT-PCR) and subsequently experienced acute respiratory failure were included in the study. Baseline characteristics, including vaccination history, along with information regarding critical illness and clinical outcomes, were gathered and compared between patients who received the vaccine and those who did not.

**Results:**

A total of 215 patients with COVID-19 exhibiting acute respiratory failure, as confirmed via RT‒PCR, were included in the analysis. Of this cohort, sixty-six (30.7%) patients died within 28 days. Neither administration of the vaccine nor achievement of primary series vaccination status had a significantly different effect on 28 day mortality, number of viral shedding events, acute respiratory distress syndrome (ARDS) incidence or other clinical outcomes. Patients who received the booster vaccine and completed the primary series showed a tendency of increased 28 days of ventilator-free status, though this difference was not statistically significant (*p* = 0.815).

**Conclusions:**

Vaccination status did not significantly influence mortality rates, the occurrence of ARDS, or the viral shedding duration in COVID-19 patients with acute respiratory failure.

## Introduction

On March 11, 2020, the World Health Organization (WHO) announced the global spread of COVID-19, a disease resulting from the severe acute respiratory syndrome coronavirus 2 (SARS-CoV-2), as a pandemic. This virus had spread across borders globally, leading to the death of millions of people. Approximately half of the people infected by the SARS-CoV-2 virus do not experience symptoms; most others experience mild to moderate symptoms. However, about 15% of patients develop severe pneumonia that requires hospital care and extra oxygen. Approximately 5% of patients develop acute respiratory distress syndrome (ARDS), multi-organ failure, or septic shock, contributing to a high mortality rate in the initial stages of the outbreak in China [1–3]. Nevertheless, the severity of the disease’s impact has changed over time, likely due to differences caused by SARS-CoV-2 mutations, the introduction of vaccination programs, and advances in treatment methods [4–6].

Complications affecting the respiratory system is the most common complication of COVID-19, and patients usually experience symptoms approximately 1 week after infection. Some of these patients develop severe hypoxemia and need respiratory support [2, 6–11]. Indeed, around one-third of patients hospitalized with COVID-19 develop ARDS, a potentially fatal manifestation of respiratory failure. Of concern, almost three-quarters of COVID-19 patients with intensive care units admission experience ARDS [8, 12–14].

To attenuate the destructive influence of viruses on public health, the economy, and societal structure, vaccines have become key strategies for controlling viral spread. As of December 20, 2021,

The WHO’s list for Emergency Use Authorization featured eight distinct COVID-19 vaccines [15]. COVID-19 vaccines, which exhibit significant efficacy in inhibiting SARS-CoV-2 infections and attenuating severe disease progression, are instrumental in decreasing hospitalization rates, decreasing disease severity, and enhancing patient outcomes [16–19]. However, the influence of vaccination on patients who develop critical illness remains a debated topic in current research. Some studies have shown that vaccinations enhance patient outcomes, including mortality rates, in critically ill patients [20, 21]. Conversely, a notable portion of studies indicate no discernible improvement in mortality after vaccination [22–28].

The objective of this study was to explore the clinical outcomes and clinical traits of COVID-19 patients with respiratory failure. Additionally, we aimed to compare clinical outcomes based on vaccination status among severe COVID-19 patients exhibiting respiratory failure.

## Methods

### Study design and participants

This retrospective cohort study was conducted at a 2800-bed tertiary medical center in northern Taiwan from April 2022 to September 2022. Patients with SARS-CoV-2 infection, as confirmed via reverse transcription polymerase chain reaction (RT‒PCR), with concomitant respiratory failure were enrolled. All enrolled patients were classified as having severe disease according to the WHO clinical progression scale [29]. Furthermore, the enrolled patients had severe or life-threatening (grade 3 or grade 4) cytokine release syndrome (CRS) resulting from a cytokine storm. Cytokine storms are systemic inflammatory processes driven by cytokines and are akin to cytokine release syndrome (CRS), a known complication of chimeric antigen receptor T-cell therapy. We adopted a widely used CRS grading system that is based on clinical symptoms [30]. The exclusion criteria included patients under the age of 20 years, those habitually reliant on a continuous positive airway pressure (CPAP) device at home, and those with a known human immunodeficiency virus infection. The COVID-19 outbreak in Taiwan during the study period was caused predominantly by the Omicron variant [31].

### SARS-CoV-2 RT‒PCR and viral shedding assessment

The Cobas^®^6800 system (Roche, Pleasanton, CA, USA) was deployed to conduct SARS-CoV-2 RT-PCR. This approach is a single-well dual target assay, encompassing specific detection of SARS-CoV-2’s ORF1ab nonstructural region and pan-Sarbecovirus detection of the conserved envelope E-gene including SARS-CoV-2. Ct values for both genes were evaluated across all RT‒PCR tests utilizing nasopharyngeal swabs or endotracheal aspirates from mechanically ventilated individuals, aligning with a previously published methodology [32]. Amidst RT-PCR tests, samples were declared positive if the Ct value was 40 or lower. Viral dynamics were analyzed via the Ct value for the SARS-CoV-2 target, ORF1ab. Viral shedding duration was determined from symptom onset until the initial increase in the Ct value above 30. Inspired by previous studies suggesting no virus isolation post Ct value exceeding 30, this metric signified a viable upper threshold with reduced transmission risk [33–37]. Additionally, a Ct value of 30 or higher was utilized to designate the deisolation threshold per Taiwan Centers for Disease Control guidelines [38]. Hospitalized patients with Ct values < 30 were right-censored at their final documented Ct value in the SARS-CoV-2 RT‒PCR report.

### Respiratory failure and ARDS

Respiratory failure was identified as the condition requiring the use of respiratory support devices. Respiratory support devices include invasive mechanical ventilation (IMV), noninvasive ventilation (NIV), and high-flow nasal cannula (HFNC) devices [39]. ARDS incidence was assessed in accordance with the Berlin definition [40] and potential modifications thereof [41, 42], outlined as follows: (1) emergent manifestation of respiratory distress within a seven-day period, (2) bilateral pulmonary opacities detected by imaging modalities, and (3) Hypoxemia was determined by a ratio of arterial oxygen partial pressure to the fraction of inspired oxygen (PaO2/FiO2) that was 300 or lower. All chest radiographs or chest computed tomography scans were reviewed by pulmonologists.

### Vaccinations

The vaccines available in Taiwan before the study period included the following: one adenoviral-vectored vaccine, ChAdOx1 nCoV-19 (University of Oxford/AstraZeneca); one subunit vaccine, MVC-COV1901 (Medigen, Taiwan); and two messenger RNA (mRNA) vaccines, mRNA-1273 (Moderna) and BNT162b2 (Pfizer-BioNTech). The number of vaccine doses was recorded. The WHO considers two doses of any WHO emergency use listing vaccine as a complete primary series. A booster dose refers to an additional vaccination given to someone who has already received a primary vaccination series [43, 44].

### Data collection and measurements

Data pertaining to demographic parameters and underlying comorbid conditions were extracted from an exhaustive review of hospital records. Laboratory test outcomes beginning on the day of respiratory insufficiency onset were diligently documented. Severity was gauged via the Acute Physiology and Chronic Health Evaluation (APACHE) II score [45] and Sequential Organ Failure Assessment (SOFA) score [46]. Records of respiratory support devices (IMV, NIV, and HFNC) use, extracorporeal membrane oxygenation (ECMO) and renal replacement therapy were meticulously maintained. Information about therapeutics associated with COVID-19, such as corticosteroids, tocilizumab, remdesivir, paxlovid, molnupiravir, and enoxaparin, was obtained. Corticosteroid administration was quantified in terms of dexamethasone equivalents and was computed based on the mean daily dosage of dexamethasone, as measured in milligrams (mg), during the period of hospitalization.

### Outcome evaluation

The primary outcome evaluated in the present study was the all-cause mortality rate on Day 28. Secondary outcomes included complications during hospitalization, duration of SARS-CoV-2 shedding, length of hospital stay, 28 day ventilatory-free days, all-cause mortality upon hospital discharge, percentage of patients developed pulmonary fibrosis secondary to cytokine storm, and the incidence of patients who acquired secondary bacterial pneumonia. All patients were followed up from admission to death or discharge.

### Statistical analysis

The Kolmogorov‒Smirnov test was performed to assess the normality of the data in our database. The findings are presented as the median with the interquartile range (IQR) or quantity (percentage), as appropriate. To compare continuous variables between two groups, the Student's t-test or the Mann–Whitney *U* test was employed. For comparisons involving continuous variables across more than two groups, either one-way ANOVA or the Kruskal–Wallis *H* test was applied. Pearson’s chi-square test was employed for categorical variables when appropriate. Logistic regression was used to calculate odds ratios (ORs) and 95% confidence intervals (CIs). Logistic regression analyses treated variables independently within the multiple logistic regression framework, applying a stepwise forward selection method. The proportions of overall mortality and viral shedding of SARS-CoV-2 were evaluated using Kaplan‒Meier survival analysis and the log-rank test. *P-*values < 0.05 were considered to indicate statistical significance. We conducted a post hoc power analysis based on the obtained sample size of 215 patients and focusing on the primary outcome of 28 day mortality, revealed a power of 0.856 in patients with ARDS at an alpha level of 0.05. All statistical analyses were performed using IBM SPSS Statistics for Windows/Macintosh, Version 25.0 (IBM Corp., Armonk, NY, USA), Prism GraphPad, Version 6.01 and MedCalc, Version 20.215.

## Results

### Patient characteristics

**Fig. 1:**
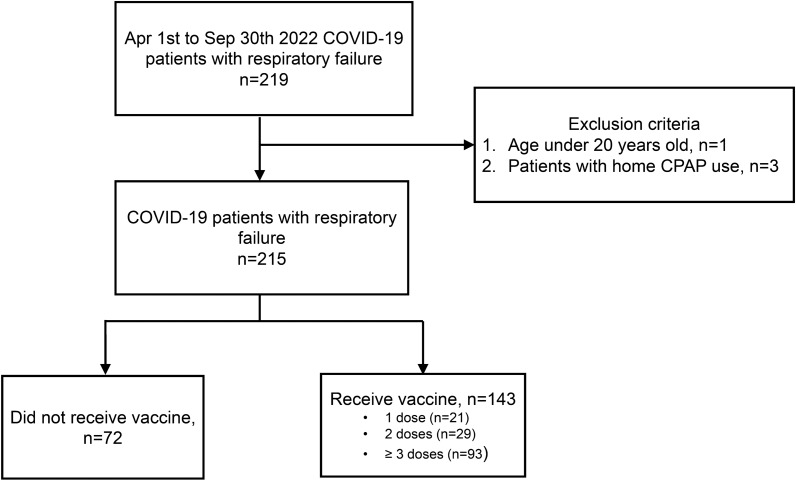
Study flowchart. *COVID-19* Coronavirus Disease 2019, *CPAP* continuous positive airway pressure

During the study period, 219 patients with RT‒PCR-confirmed COVID-19 accompanied by respiratory failure were eligible for inclusion. After excluding 3 patients with chronic home-based CPAP and 1 patient under the age of 20 years, the analysis included a cohort of 215 patients (Fig. 1). The demographic parameters and clinical characteristics of the patient cohort at enrollment are shown in Table 1. One hundred forty-three (66.5%) patients were administered a minimum of one dose of vaccine, sixty-six (30.7%) patients died within 28 days, and 147 (68.4%) met the ARDS criteria at the time of respiratory failure diagnosis.

**Table 1:** Clinical characteristics of patients with COVID-19 categorized according to vaccination status

	All cases (*n* = 215)	Vaccination (*n* = 143)	Non vaccination (*n* = 72)	*p*-value
Demographics				
Age, year	80 (67–89)	80 (68–87)	80 (64–90)	0.738
Male	145 (67.4)	103 (72.0)	42 (58.3)	0.043
Body mass index, kg/m^2^	21.9 (18.8–24.9)	21.9 (19.1–24.9)	21.6 (18.7–24.9)	0.362
Underlying disease				
Cerebrovascular disease	37 (17.2)	29 (20.3)	8 (11.1)	0.093
Dementia	31 (14.4)	19 (13.3)	12 (16.7)	0.506
Heart failure	22 (10.2)	14 (9.8)	8 (11.1)	0.763
Diabetes mellitus	86 (40)	56 (39.2)	30 (41.7)	0.723
Chronic kidney disease	50 (23.3)	36 (25.2)	14 (19.4)	0.348
Hepatobiliary disease	22 (10.2)	14 (9.8)	8 (11.1)	0.763
Uncured malignancy	65 (30.2)	45 (31.5)	20 (27.8)	0.578
Immunocompromised	83 (38.6)	57 (39.9)	26 (36.1)	0.594
Laboratory data				
White blood cells, 10^9^/L	11150 (7015–15740)	10910 (7255–15945)	11270 (5637–15688)	0.624
Lymphocytes, 10^8^/L	702 (440–1214)	749 (456–1275)	679 (408–1124)	0.498
Albumin, g/dL	3.1 (2.7–3.4)	3.1 (2.6–3.5)	3.0 (2.7–3.4)	0.467
C-reactive protein, mg/dL	6.1 (1.9–13.8)	6.0 (2.1–14.0)	6.6 (1.8–13.7)	0.921
Procalcitonin, ng/mL	0.72 (0.24–3.24)	0.7 (2.2–3.1)	0.7 (2.3–3.9)	0.532
LDH, U/L	363 (240–500)	346 (237–499)	368 (256–525)	0.459
Lactate, mg/dL	23.3 (14.8–39.1)	23.3 (14.2–39.3)	24.0 (15.7–33.7)	0.955
D-dimer, ug/mL	2.45 (1.19–5.68)	2.35 (1.19–5.66)	2.59 (1.19–6.38)	0.725
Fibrinogen, mg/dL	381 (314–514)	378 (303–514)	404 (322–507)	0.607
Initial severity				
ARDS	147 (68.4)	98 (68.5)	49 (68.1)	0.944
SOFA	8 (6–11)	8 (5–11)	8 (6–11)	0.901
APACHEII	24 (18–29)	24(17–29)	23 (19–29)	0.938
PaO2/FiO2	140 (88–239)	139 (89–248)	144 (83–233)	0.453
Ventilation				
Invasive mechanical ventilation	131 (60.9)	87 (60.8)	44 (61.1)	0.969
NIPPV	18 (8.4)	11 (7.7)	7 (9.7)	0.612
HFNC	87 (40.5)	59 (41.3)	28(38.9)	0.738
ECMO	8 (3.7)	6 (4.2)	2 (2.8)	0.604
Treatment				
Tocilizumab	76 (35.3)	51 (35.7)	25 (34.7)	0.892
Remdesivir	169 (78.6)	108 (75.5)	61 (84.7)	0.121
Paxlovid	6(2.8)	1 (0.7)	5 (6.9)	0.008
Molnupiravir	11 (5.1)	7 (4.9)	4 (5.6)	0.836
Enoxaparin	72 (33.5)	443 (0.8)	28 (38.9)	0.234
Mean daily steroid dose	5.6 (6.0–6.8)	6.0 (5.6–6.7)	6.0 (5.6–7.2)	0.580
Newly renal replacement therapy	21 (9.8)	15 (10.5)	6 (8.3)	0.615
Initial vasopressor	70 (32.6)	45 (31.5)	25 (34.7)	0.631

Vaccination means receiving at least one dose of COVID-19 vaccine. Mean daily steroid dose means average dexamethasone dose per day within whole hospitalization. Continuous data are expressed as median with IQR, and categorical data are expressed as number of patients (%). *P*-value is analyzed by Chi-square test, Student’s *t*-test, or Mann–Whitney U test. Data are presented as median (IQR) and *n*(%) unless otherwise indicated. *N/A* not applicable

*LDH* lactate dehydrogenase, *ARDS* acute respiratory distress syndrome, *SOFA* Sequential Organ Failure Assessment, *APACHE II* Acute Physiology and Chronic Health Evaluation, *ECMO* extracorporeal membrane oxygenation

### Influence of vaccination on clinical outcomes

**Fig. 2:**
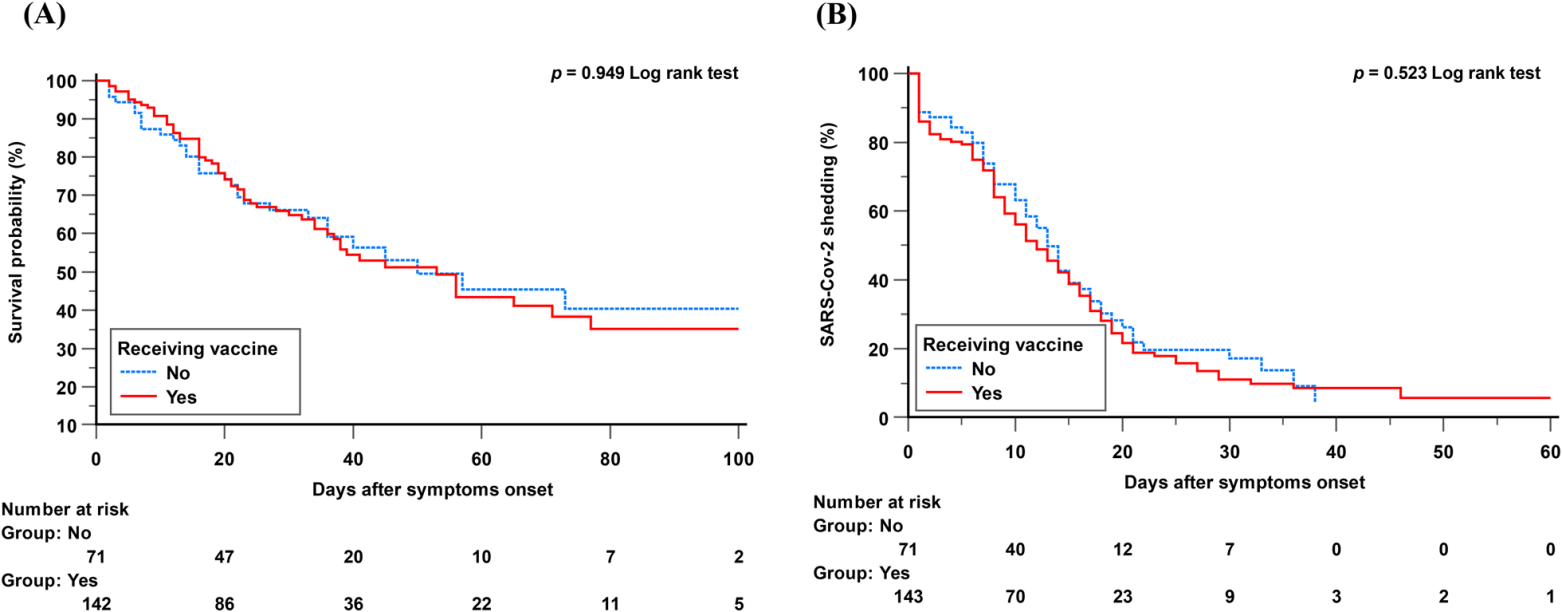
K‒M curves for the cumulative portion of events based on vaccine status. **A** Survival probability did not significantly differ between those who were vaccinated and those who were not (log-rank test: *p* = 0.949). **B** There wasn’t a notable disparity in the proportion of patients displaying SARS-CoV-2 RNA Ct values below 30 within 30 days of the onset of illness, regardless of their vaccination status (log-rank test: *p* = 0.523)

Table 1 shows the comparative demographic parameters and clinical attributes between patients who received the vaccine and those who did not. There was no significant difference in patient characteristics between patients who received the vaccine and those who did not. Comparisons of complications and clinical outcomes are shown in Table 2. The vaccination status did not significantly affect 28 day mortality, the occurrence of ARDS, the duration of viral shedding or other complications or clinical outcomes. As shown in Fig. 2, survival analysis indicated that neither the mortality rate during hospitalization nor the time from symptom onset to achieving a Ct value greater than 30 differed significantly between groups based on vaccination status.

**Table 2:** Clinical outcomes of patients with COVID-19 categorized according to vaccination status

	All cases (*n* = 215)	Vaccination (*n* = 143)	Non-vaccination (*n* = 72)	*p*-value
Outcomes				
In hospital mortality	95 (44.2)	63 (44.1)	32 (44.4)	0.957
28-day mortality	66 (30.7)	43 (30.1)	23 (31.9)	0.779
Hospital days	27 (16–42)	25 (15–41)	30 (16–43)	0.509
28-day ventilator-free days	4 (0–18)	6 (0–17)	2 (0–20)	0.917
Time from symptoms onset to 1st Ct > 30, days	11 (5–17)	10 (5–17)	11 (6–18)	0.575
Complications				
CMV infection	38 (17.7)	27 (18.9)	11 (15.3)	0.686
GI bleeding	62 (28.8)	45 (31.5)	17 (23.6)	0.230
Other bleeding	50 (23.3)	38 (26.6)	12 (16.7)	0.105
Barotrauma	7 (3.3)	6 (4.2)	1 (1.4)	0.274
Thrombosis	13 (6.0)	10 (7.0)	3 (4.2)	0.412
Secondary bacterial pneumonia	108 (50.2)	70 (49.0)	38 (52.8)	0.596
Pulmonary fibrosis	13 (37.1)	9 (39.1)	4 (33.3)	0.736

Vaccination means receiving at least one dose of COVID-19 vaccine. Data are presented as median (IQR) and *n*(%) unless otherwise indicated. Continuous data are expressed as median with IQR, and categorical data are expressed as number of patients (%). *P*-value is analyzed by Chi-square test or Mann–Whitney *U* test

*CMV* cytomegalovirus

**Fig. 3:**
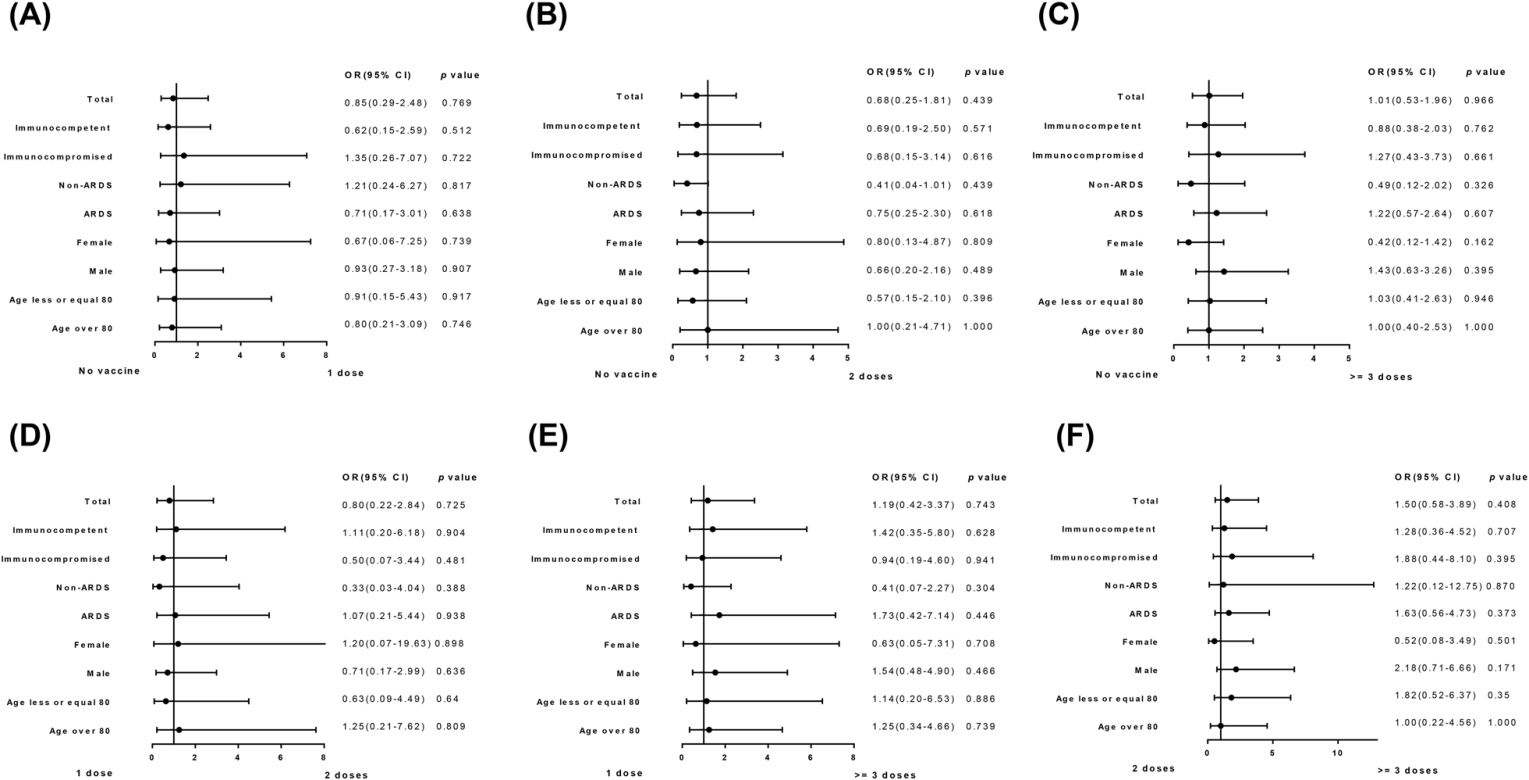
Forest plot analysis for the association between vaccine doses and 28 day mortality according to subgroups. Comparison of odds ratios of 28 day mortality between different subgroup based on vaccine doses. Age and body mass index were stratified according to median of the total population. **A** Received one dose of vaccine vs. did not receive vaccine. **B** Received two doses of vaccine vs. did not receive vaccine. **C** Received at least three doses of vaccine vs. did not receive vaccine. **D** Received two doses of vaccine vs. receive one dose of vaccine. **E** Received at least three doses of vaccines vs. receive one dose of vaccine. **F** Received at least three doses of vaccines vs. receive two doses of vaccine

**Fig. 4:**
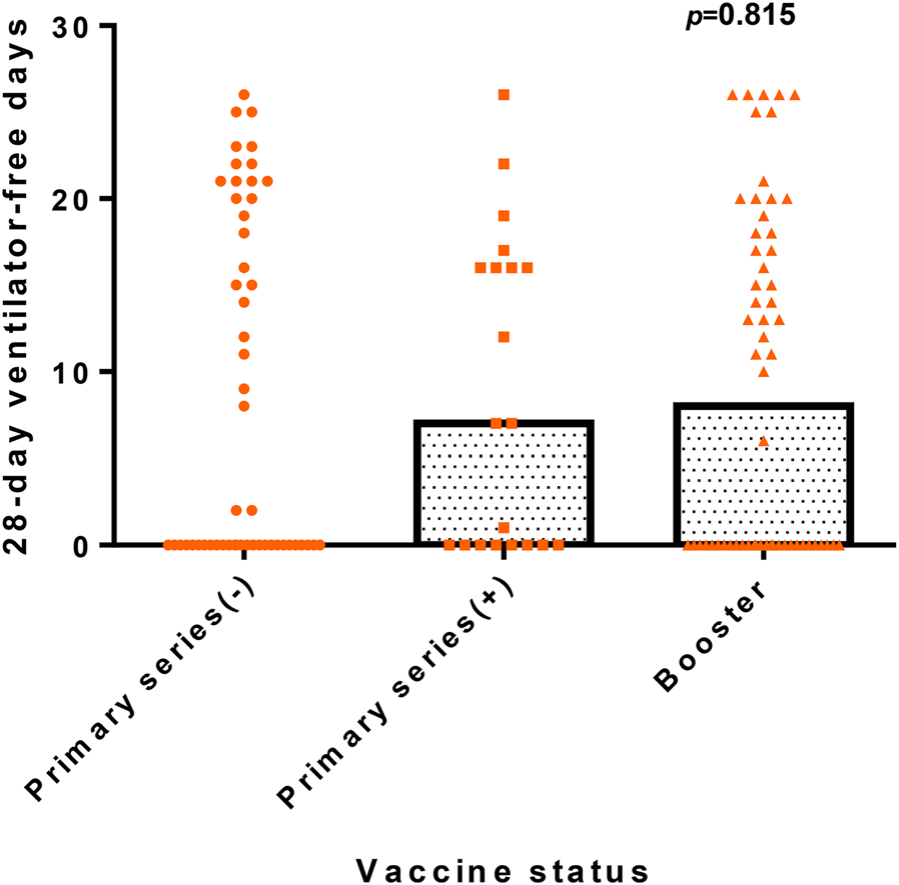
Distribution of 28 day ventilator-free days categorized according to vaccine status. The primary series (− ) group included patients who did not complete the primary vaccine series (0 or 1 dose), the primary series ( +) group included patients who completed the primary vaccine series (2 doses), and the booster group included patients who received the booster vaccine (3 or more doses). The bar chart in the figure indicates the median number of 28 day ventilator-free days. The *p*-value was determined by the Kruskal–Wallis *H* test

As shown in Fig. 3, findings were consistent across all the subgroups of the study cohort, including those stratified by sex, immune status, ARDS status, and age, as well as across various comparisons of vaccine doses. When the entire population was divided into three groups—not completing the primary vaccine series (0 or 1 dose), completing the primary vaccine series (2 doses), and receiving the booster vaccine (3 or more doses)—a trend emerged showing that individuals who received the booster vaccine and those who completed the primary series had more 28 day ventilator-free days than those who did not complete the primary series (Table 3 and Fig. 4). However, this trend was not statistically significant (*p* = 0.815). Moreover, there was no significant difference in 28 day mortality or other clinical outcomes.

**Table 3:** Severity and outcomes of patients with COVID-19 categorized according to primary series dose of vaccine

	Primary series (− ) (*n* = 91)	Primary series ( +) (*n* = 29)	Booster doses (*n* = 93)	*p*-value
Severity				
PaO2/FiO2 ratio	141 (82–135)	140 (105–234)	139 (84–250)	0.747
SOFA	8 (6–11)	10 (5–12)	8 (6–11)	0.560
APACHEII	23 (19–28)	24 (18–32)	25 (17–29)	0.883
ARDS	60 (64.5)	21 (72.4)	66 (71.0)	0.563
Life support				
Mechanical ventilation	53 (57.0)	20 (69.0)	58 (62.4)	0.479
Newly renal replacement therapy	8 (8.6)	2(6.9)	11 (11.8)	0.650
ECMO	2 (2.2)	3 (10.3)	3 (3.2)	0.119
Initial vasopressors use	31 (33.3)	8 (27.6)	31 (33.3)	0.828
Outcomes				
Hospital days	30 (14–45)	23 (13–43)	25 (16–39)	0.737
28-day ventilator-free days	0 (0–19)	7 (0–16)	10 (0–18)	0.879
In hospital mortality	41 (44.1)	11 (37.9)	43 (46.2)	0.734
28-day mortality	29 (31.2)	7 (24.1)	30 (32.3)	0.704
Time from symptoms onset to 1st Ct > 30, days	11 (6–17)	12 (6–18)	9 (1–18)	0.675

Primary series (− ): not completing the primary vaccine series (0 or 1 dose). Primary series ( +): completing the primary vaccine series (2 doses). Booster doses: receiving the booster vaccine (3 or more doses). Continuous data are expressed as median with IQR, and categorical data are expressed as number of patients (%). *P*-value is analyzed by Chi-square test, one way ANOVA or Kruskal–Wallis *H* test. Data are presented as median (IQR) and *n*(%) unless otherwise indicated. *N/A* not applicable

*SOFA* Sequential Organ Failure Assessment, *APACHE II* Acute Physiology and Chronic Health Evaluation, A*R*DS acute respiratory distress syndrome, *ECMO* extracorporeal membrane Oxygenation

### Risk factors for 28-day mortality

Compared with surviving patients, those who died within a 28 day period had higher levels of procalcitonin, lactate dehydrogenase (LDH), and D-dimer; more acute respiratory syndrome (ARDS); more need for vasopressor use; and fewer ventilator-free days. Additionally, patients who died within the 28 day timeframe exhibited longer SARS-CoV-2 shedding. However, IMV was used more often in surviving patients, possibly because a greater number of patients who did not survive had ‘Do-Not-Resuscitate’ decisions and might have chosen not to undergo intubation. Univariate and multivariate examinations of clinical factors related to 28 day mortality are presented in Table 4. Univariate analysis indicated that clinical factors correlated with 28 day mortality, including initial ARDS incidence, increased LDH and D-dimer levels, initial vasopressor use, and increased APACHE II score. Multivariate analysis revealed that the independent variables linked with 28 day mortality were higher LDH and D-dimer levels and initial ARDS (aOR, 2.520; 95% CI 1.015–6.257).

**Table 4:** Factors associated with 28-day mortality by multivariate and univariate logistic regression analysis

	Univariate			Multivariate		
	Odds ratio	95% CI	*p*-value	Odds ratio	95% CI	*p*-value
Initial ARDS	2.111	1.07–4.16	0.031	2.520	1.015–6.257	0.046
Vaccination	0.916	0.50–1.69	0.779			
Age	1.010	0.99–1.03	0.326			
Procalcitonin	1.011	0.99–1.03	0.243			
LDH	1.001	1.00–1.00	0.040	1.001	1.0002–1.0027	0.023
D-dimer	1.035	1.01–1.06	0.007	1.039	1.009–1.070	0.011
Initial vasopressor	1.877	1.03–3.44	0.041			
APACHE II	1.038	1.001–1.076	0.045			

Vaccination means receiving at least one dose of COVID-19 vaccine

*CI* confidence interval, *LDH* lactate dehydrogenase, *APACHE II* Acute Physiology and Chronic Health Evaluation

### Influence of initial ARDS onset on clinical outcomes

Survival analysis revealed a markedly increased incidence of mortality over the course of hospitalization (log-rank *p* = 0.038), along with significantly longer time intervals from symptom emergence to Ct > 30 in patients with initial ARDS (log-rank *p* = 0.0498).

## Discussion

Although there appears to be a pattern indicating that individuals with the booster vaccine and those who completed the primary series experience more 28 day ventilator-free days, vaccination status was not significantly related to outcomes in critical patients with COVID-19 in our study. Our research conclusively substantiates that initial ARDS is independently linked to the 28 day mortality rate.

In our investigation, ARDS displayed a profound association with the 28 day mortality rate and overall hospital mortality, a finding consistent with previous studies [47, 48]. Our study found that patients with initial ARDS had a longer period of viral shedding compared to those without ARDS. A higher viral load of SARS-CoV-2 is a factor consistently associated with increased disease severity and worse prognosis [49, 50]. Extended viral shedding duration has been documented among critically ill patients and has shown a profound correlation with their ultimate survival outcomes [32]. In some research, bilateral pulmonary infiltrates and respiratory failure have been associated with prolonged viral shedding [32, 51]. A reasonable hypothesis is that the occurrence of ARDS is a sign of disease severity, resulting from the excessive inflammatory response triggered by COVID-19 [52]. Such a response may culminate in immune dysregulation, an element postulated to be a potential catalyst in relation to protraction of SARS-CoV-2 shedding [36, 53, 54].

Our results showed that vaccination was not significantly related to mortality, a conclusion that aligns with most published studies discussing the effect of vaccines in critically ill COVID-19 patients [22–28]. Additionally, our analysis revealed clinical differences and variations in outcomes across different vaccine doses and among various patient subgroups**.** However, none of these analyses revealed a significant impact due to vaccination. Several hypotheses may explain our findings. First, the effect of the vaccine varies among individuals [55], and COVID-19 vaccination can lead to reduced immunogenicity in individuals with a spectrum of immune conditions [56–59]. Disease severity, as marked by respiratory failure, may suggest different immune statuses than in people who are not critically ill. Consequently, the effect of the vaccine on clinical outcomes may also exhibit variance.

According to our findings, the clinical efficacy of vaccines may be diminished in patients with respiratory failure. Thus, it is vital to initiate early and optimal treatment before patients’ progress to respiratory failure. This includes use of steroids, antiviral agents, and proper supportive care [6]. Despite the lack of evidence for reducing mortality in critically ill COVID-19 patients, our findings indicate that vaccination potentially reduces the duration of ventilator use. According to other recent studies, even if vaccines do not lower mortality rates, they may offer other clinical benefits to critically ill patients [26, 27]. Additionally, considering other known benefits, such as decreasing hospitalization rates and disease severity, use of COVID-19 vaccines should be actively promoted.

Our study has few limitations. First, due to its design as a retrospective cohort study, some important data might remain undiscovered. Second, the lack of a protocolized screening for neutralizing antibody levels impedes adequate evaluation of the immunity conferred by vaccination among our cohort of patients with COVID-19. The study period spanned from April 2022 to September 2022, during which the genetic variants of SARS-CoV-2 and the vaccine underwent alterations. Therefore, the applicability of these findings to the present scenario remains uncertain. However, our study also had several strengths. First, we evaluated the clinical outcomes of patients with respiratory failure, including the duration of viral shedding. This aspect might indicate the immune status, serving as a clinical outcome potentially linked to vaccination [60]. Second, we thoroughly analyzed the influence of the vaccine on clinical outcomes across various vaccine doses and among different patient subgroups. To gain a more comprehensive understanding of the relationship between vaccination and clinical outcomes in critically ill patients with COVID-19, a well-designed prospective clinical study is required.

## Conclusions

Vaccination status has no significant influence on mortality rates, the occurrence of ARDS, or the duration of viral shedding in COVID-19 patients with acute respiratory failure. Initial ARDS is associated with the 28 day mortality.

## Data Availability

The datasets used and/or analyzed during the current study are available from the corresponding author upon reasonable request.

## Abbreviations

APACHE: Acute Physiology and Chronic Health Evaluation
ARDS: Acute respiratory distress syndrome
CIs: Confidence intervals
COVID-19: Coronavirus Disease 2019
CPAP: Continuous positive airway pressure
CRS: Cytokine release syndrome
Ct: Cycle threshold
ECMO: Extracorporeal membrane oxygenation
HFNC: High-flow nasal cannula
WHO: World Health Organization
IMV: Invasive mechanical ventilation
IQR: Interquartile range
LDH: Lactate dehydrogenase
Mg: Milligrams
mRNA: Messenger RNA
NIV: Noninvasive ventilation
ORs: Odds ratios
PaO2/FiO2: Ratio of arterial oxygen partial pressure to fraction of inspired oxygen
SARS-CoV-2: Severe Acute Respiratory Syndrome Coronavirus 2
SOFA: Sequential Organ Failure Assessment
RT‒PCR: Reverse transcription polymerase chain reaction
